# Observation of a re-entrant phase transition in the molecular complex tris(μ_2_-3,5-diiso­propyl-1,2,4-triazolato-κ^2^ *N* ^1^:*N* ^2^)trigold(I) under high pressure

**DOI:** 10.1107/S2052252516013129

**Published:** 2016-08-18

**Authors:** Christopher H. Woodall, Jeppe Christensen, Jonathan M. Skelton, Lauren E. Hatcher, Andrew Parlett, Paul R. Raithby, Aron Walsh, Stephen C. Parker, Christine M. Beavers, Simon J. Teat, Mourad Intissar, Christian Reber, David R. Allan

**Affiliations:** a Department of Chemistry, University of Bath, Bath BA2 7AY, UK; b Research Complex at Harwell, Rutherford Appleton Laboratory, Harwell Oxford, Didcot, Oxfordshire, OX11 0FA, UK; c Station 11.3.1, Advanced Light Source, Lawrence Berkeley National Laboratory, Berkeley, CA 94720, USA; d Département de Chimie, Université de Montréal, Montréal, Québec, H3C 3J7, Canada; e Station I19, Diamond Light Source, Didcot, Oxfordshire, OX11 0QX, UK

**Keywords:** re-entrant phase transitions, high-pressure crystallography, gold(I), luminescence spectroscopy, DFT calculations

## Abstract

We report a molecular crystal that exhibits four successive phase transitions under hydro­static pressure, driven by aurophilic interactions, with the ground-state structure re-emerging at high pressure. The effect of pressure on two polytypes of tris(μ_2_-3,5-diiso­propyl-1,2,4-triazolato-κ^2^ *N* ^1^:*N* ^2^)trigold(I) (denoted Form-I and Form-II) has been analysed using luminescence spectroscopy, single-crystal X-ray diffraction and first-principles computation. A unique phase behaviour was observed in Form-I, with a complex sequence of phase transitions between 1 and 3.5 GPa. The ambient *C*2/*c* mother cell transforms to a *P*2_1_/*n* phase above 1 GPa, followed by a *P*2_1_/*a* phase above 2 GPa and a large-volume *C*2/*c* supercell at 2.70 GPa, with the previously observed *P*2_1_/*n* phase then reappearing at higher pressure. The observation of crystallographically identical low- and high-pressure *P*2_1_/*n* phases makes this a rare example of a re-entrant phase transformation. The phase behaviour has been characterized using detailed crystallographic theory and modelling, and rationalized in terms of molecular structural distortions. The dramatic changes in conformation are correlated with shifts of the luminescence maxima, from a band maximum at 14040 cm^−1^ at 2.40 GPa, decreasing steeply to 13550 cm^−1^ at 3 GPa. A similar study of Form-II displays more conventional crystallographic behaviour, indicating that the complex behaviour observed in Form-I is likely to be a direct consequence of the differences in crystal packing between the two polytypes.

## Abstract 2

Single-crystal crystallography shows that one polytype of the Au^I^ trimer tris(μ_2_-3,5-diiso­propyl-1,2,4-triazolato-κ^2^ *N* ^1^:*N* ^2^)trigold(I), [Au_3_(N_3_C_8_H_14_)_3_], undergoes four successive phase transitions between 1 and 3.5 GPa, driven by aurophilic interactions, and that the ambient-pressure structure re-emerges at high pressure.

## 1. Introduction   

Gold(I) trimers are an intriguing class of compounds, attracting attention due to their unique photophysical properties (Yam & Cheng, 2007 ▸, 2008 ▸), including solvochromism (Fung *et al.*, 1998 ▸; Vickery *et al.*, 1997 ▸) and thermochromism (Yang *et al.*, 2006 ▸). These properties are typically associated with the presence of intermolecular aurophilic interactions in the solid state, which leads to aggregation into more complex and unusual supramolecular geometries (White-Morris *et al.*, 2005 ▸; Burini *et al.*, 2003 ▸; Schmidbaur & Schier, 2012 ▸). These interactions are easily perturbed through changes in environmental conditions, such as temperature or exposure to solvent.

Hydro­static pressure is an environmental variable that has not been utilized extensively to investigate the physical and electronic properties of gold(I) trimers. It has been demonstrated that high hydro­static pressure can be an effective tool for altering molecular geometries and hence the tuning of molecular properties in a variety of molecular materials, including energetic materials (Fabbiani & Pulham, 2006 ▸; Millar *et al.*, 2010 ▸), molecular magnets (Parois *et al.*, 2010 ▸; Prescimone, Milios, Moggach, Warren, Lennie, Sanchez-Benitez* et al.*, 2008 ▸; Prescimone, Sanchez-Benitez* et al.*, 2009 ▸; Prescimone, Milios* et al.*, 2009 ▸; Prescimone, Milios, Moggach, Warren, Lennie, Sanchez-Benitez* et al.*, 2008 ▸; Tancharakorn *et al.*, 2006 ▸), spin crossover systems (Shepherd *et al.*, 2011 ▸, 2012 ▸) and metal–organic frameworks (Moggach *et al.*, 2009 ▸; Fairen-Jimenez *et al.*, 2011 ▸), as well as a limited number of gold systems (Paliwoda *et al.*, 2014 ▸; Roberts *et al.*, 2014 ▸; Baril-Robert *et al.*, 2012 ▸; Cairns *et al.*, 2013 ▸). In addition to its effect on a range of properties, hydro­static pressure is a unique tool for inducing unexpectedly complex phase behaviour in relatively simple materials, such as elemental metals (Nelmes *et al.*, 1999 ▸; Loa *et al.*, 2012 ▸; McMahon & Nelmes, 2006 ▸), one of the more spectacular examples being the commensurate host–guest structure of Ba at 19 GPa reported by Loa *et al.* (2012 ▸). The observation of such complex phase behaviour has been attributed to the fact that high-pressure forces electron density to order in less conventional ways to accommodate the pressure-induced reduction in volume, and hence minimize the impact of the *pV* contribution to the enthalpy of the system. A limited number of other elemental metals (Porsch & Holzapfel, 1993 ▸), as well as peroskites and pure inorganics (Kabbour *et al.*, 2012 ▸; Downie *et al.*, 2013 ▸; Brinkmann *et al.*, 2004 ▸), have been show to display pressure-induced re-entrant phase behaviour, defined by Dove as a transition where there is a sequence of two phase transitions, with the first and third phases having the same symmetry and effectively identical structures (Dove, 2011 ▸). In molecular solids, such transitions are exceptionally rare, with only two well-known purely structural examples, namely rochelle salt and malo­nitrile (Levitskii *et al.*, 2010 ▸; Beevers & Hughes, 1941 ▸; Suzuki & Shiozaki, 1996 ▸; David *et al.*, 2013 ▸; Dove & Rae, 1983 ▸; Wasiutynski *et al.*, 1987 ▸; Dove, 2011 ▸). There are also a few examples of spin crossover materials that have been identified to have re-entrant phases, driven by the electronic transitions from a high-spin structure to a low-spin structure *via* an intermediate phase (Chernyshov *et al.*, 2003 ▸). In all these cases, the re-entrant transition occurs with variation in temperature rather than pressure.

**Figure 1:**
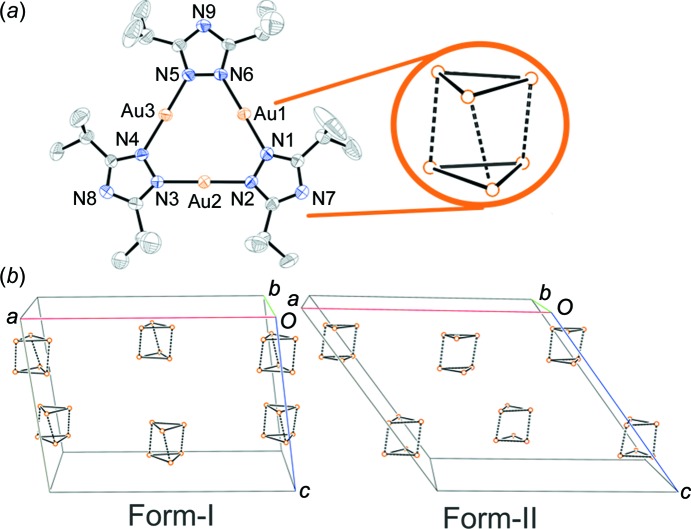
(*a*) The X-ray structure of tris­(μ_2_-3,5-diiso­propyl-1,2,4-triazolato-κ^2^ *N* ^1^:*N* ^2^)trigold(I) in the literature-reported phase (Form-I), with ellipsoids drawn at the 30% probability level and H atoms omitted for clarity. The inset illustrates the aggregation of the complexes in the solid state to form a staggered prismatic hexamer *via* aurophilic interactions. (*b*) The packing and aurophilic interactions in the crystal structure of Form-I (left) and Form-II (right); all atoms except gold have been omitted for clarity.

Recent reports have highlighted the changes in physical and luminescence properties of a series of gold(I) complexes where noncovalent aurophilic interactions play a key role (Tiekink, 2014 ▸; Woodall *et al.*, 2014 ▸). In this paper, we report a comprehensive investigation of the properties of tris(μ_2_-3,5-diiso­propyl-1,2,4-triazolato-κ^2^ *N* ^1^:*N* ^2^)trigold(I) under hydro­static pressures ranging from ambient to 3.31 GPa. The ther­mo­chromic behaviour of tris(μ_2_-3,5-diiso­propyl-1,2,4-tri­az­ol­ato-κ^2^ *N* ^1^:*N* ^2^)trigold(I) has been established previously, being reported by Yang *et al.* (2006 ▸) to display a shift in emission wavelength upon cooling from *∼*725 nm at 280 K to 755 nm at 90 K (λ_ex_ = 280 nm). The shift in emission is attributed to a reduction in an intermolecular aurophilic interaction length from 3.42 Å at room temperature to 3.19 Å at 90 K. Time-resolved photocrystallographic studies of the copper analogue have highlighted similar structural distortions in the excited state, confirming the link between the temperature-induced structural distortion and the change in the emission spectrum (Vorontsov *et al.*, 2005 ▸). The initial aim of the study was to compare the effect of pressure and temperature on the structure and luminescence of the complex, building on the work by various groups relating pressure-induced structural changes with dramatic shifts in the luminescence emissions in other gold(I) complexes (Paliwoda *et al.*, 2014 ▸; Roberts *et al.*, 2014 ▸; Baril-Robert *et al.*, 2012 ▸). However, upon initial experiment, the complex was discovered to display an unusual form of polytypism, that is, a near isomorphic crystal structure with differences only in layer stacking (Fig. 1 ▸), and one form of this exhibited unique re-entrant phase behaviour going through four pressure-induced single-crystal-to-single-crystal phase transitions over the pressure range from ambient to 3.5 GPa. This unique behaviour can be attributed to features within the crystal structures, and these findings have been corroborated by detailed computational and spectroscopic studies.

## 2. Experimental   

### 2.1. Synthesis   

All reagents were used as received from commercial suppliers. Tris(μ_2_-3,5-diiso­propyl-1,2,4-tri­az­ol­ato-κ^2^ *N* ^1^:*N* ^2^)tri­gold(I) was synthesized following the literature procedure of Yang *et al.* (2006 ▸). Crystals suitable for X-ray diffraction were grown *via* tetrahydro­furan (THF)/ether vapour diffusion or by slow evaporation of THF or di­chloro­methane. Crystals of Form-I and Form-II were distinguished through visual inspection and single-crystal X-ray diffraction.

### 2.2. High-pressure crystallography   

High-pressure single-crystal X-ray diffraction experiments were performed on a three-circle Bruker APEXII CCD diffractometer at station 11.3.1 of the Advanced Light Source, Lawrence Berkeley National Labs, USA, or at beamline I19 at the Diamond Light Source, Didcot, UK, using a Rigaku Saturn CCD diffractometer. A Merrill–Bassett diamond anvil cell (DAC) was used for the high-pressure measurements using Boehler–Almax diamonds with 600 µm culets. Laser-cut tungsten or steel (200 µm thickness) was used as the gasket material. Gasket holes were drilled using an Oxford Lasers laser mill to 200 µm diameter. Loading of the cell was performed for all samples using a 4:1 methanol/ethanol mix as a hydro­static medium and ruby powder as the pressure calibrant. Pressure calibration was performed *via* the ruby-fluorescence method (Piermarini *et al.*, 1975 ▸). High-pressure data was integrated using the *APEX2* (Bruker, 2005 ▸) software suite. Shielding of the diffraction pattern by the DAC was dealt with by the generation of dynamic masks using an external program (Dawson *et al.*, 2004 ▸). Each pressure point was collected in multiple cell orientations to increase data completeness and merged in *XPREP* (Bruker, 2005 ▸). A multi-scan absorption correction was performed using *SADABS* (Bruker, 2005 ▸). Data was refined against a previously determined room-temperature structure by full-matrix least-squares on *F* ^2^ using *SHELXL97* (Sheldrick, 2008 ▸). All C—C and C—N bond lengths in the structure were restrained to the values of the room-temperature structure under the assumption that such interactions are relatively resilient to compression. Metal–metal, metal–C and metal–N interactions were refined freely.

### 2.3. Luminescence measurements   

Luminescence spectra were measured with a Renishaw Invia Raman-imaging microscope equipped with a Peltier-cooled CCD camera. Excitation sources for the luminescence experiments were a 488 nm argon-ion laser and a 514 nm diode laser. The microscope was used to focus light onto a sample spot of approximately 1 µm in diameter and to collect the scattered light. Pressure-dependent measurements on solid samples in Nujol were made with a DAC (High-Pressure Diamond Optics). The ruby R1 line method (Piermarini *et al.*, 1975 ▸) was used to calibrate the hydro­static pressure inside the gasketed cell. All pressure-induced phenomena reported in this work are reversible upon gradual release of external pressure.

### 2.4. Computational studies   

Periodic first-principles calculations were carried out on the ambient *C*2/*c* and high-pressure *P*2_1_/*n* and *P*2_1_/*a* phases using the Kohn–Sham density-functional theory scheme (Kohn & Sham, 1965 ▸), as implemented in the Vienna Ab Initio Simulation Package (VASP) code (Kresse & Hafner, 1993 ▸). The PBEsol exchange-correlation functional (Constantin *et al.*, 2008 ▸) was used with a 500 eV kinetic energy cut-off for the plane–wave basis, and core electrons were treated using scalar-relativistic projector augmented-wave (PAW) pseudo­potentials (Blöchl, 1994 ▸; Kresse & Joubert, 1999 ▸). In all three crystallographic cells, the Brillouin zone was sampled at the Γ point.

## 3. Results and discussion   

### 3.1. High-pressure crystallographic studies   

Tris(μ_2_-3,5-diiso­propyl-1,2,4-tri­az­ol­ato-κ^2^ *N* ^1^:*N* ^2^)tri­gold(I) was obtained *via* the synthetic procedure reported by Yang *et al.* (2006 ▸) and recrystallization of the material yielded crystals of the monoclinic *C*2/*c* phase reported by Yang *et al.* (2006 ▸) suitable for high-pressure single-crystal diffraction experiments and high-pressure luminescence studies. Crystals of a previously unreported polytype were also obtained (Fig. 1 ▸).

The new polytype crystallizes in a monoclinic *C*2/*c* cell (Form-II), with the most significant difference between the two cells being the larger *β* angle of 121.1° in Form-II compared to the 97.3° angle in Form-I. In both forms, it can be seen that the complex crystallizes as an aurophilically bound staggered prismatic hexamer (Fig. 1 ▸), forming a layered structure, stacking along the crystallographic *c* axis to make up Form-I. Structurally, the larger offset of the hexamers of one layer relative to the layer below results in a larger *β* angle in Form-II and a marginally smaller unit-cell volume under ambient conditions (∼61 Å^3^).

High-pressure single-crystal X-ray diffraction experiments were carried out on both Form-I and Form-II, with compression of Form-I performed from ambient to 3.30 GPa and of Form-II from ambient to 2.88 GPa, in order to compare the high-pressure structural behaviour of the two polytypes. Form-I displays some remarkable phase behaviour, remaining in the ambient-pressure *C*2/*c* phase up to ∼1 GPa, where the appearance of black lines across the [001] face of the crystal were observed; these lines correspond to the cleavage planes in the crystal (see Fig. S1 ▸ in the *Supporting information*). Release of the pressure from ∼1 GPa to ambient resulted in the disappearance of these lines, but an increase in pressure above 1 GPa typically resulted in permanent deformation along the lines and a loss of coherent diffraction (*Supporting information*). On occasion, a crystal of Form-I would survive the transition to pressures above 1 GPa, remaining a coherent single crystal and displaying complex phase behaviour. Visual inspection to identify crystals with minimal defects was required to select the crystals on which to carry out high-pressure studies.

**Figure 2:**
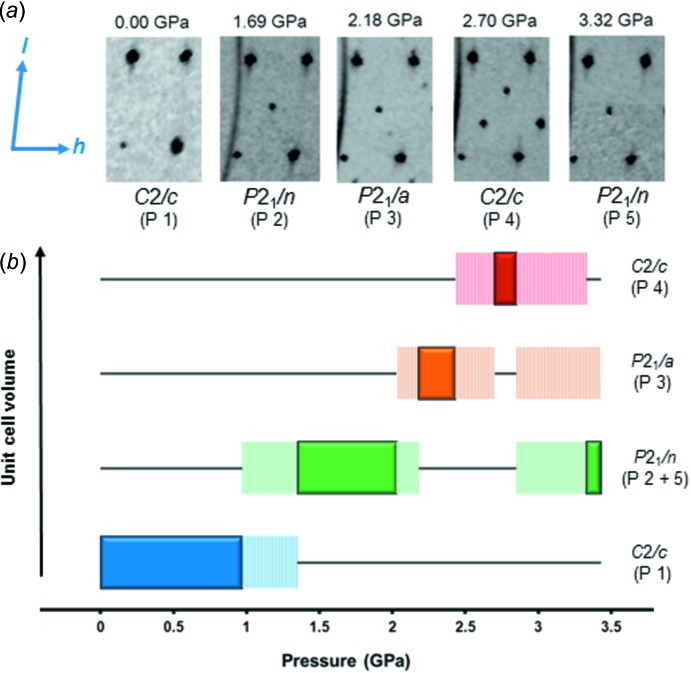
(*a*) Sections of the *h*0*l* plane of the diffraction patterns of the five observed phases of Form-I. The 12,0,0 reflection of the original *C*2/*c* mother cell occupies the top left corner. (*b*) Schematic phase diagram of Form-I as a function of pressure. The filled blocks represent experimentally recorded data, while the dashed lines indicate the intermediate co-existence of the two phases.

Above 1 GPa a transition to a new phase was observed (denoted P 2) and was apparent in the [010] planes of the diffraction pattern. The appearance of a series of Bragg peaks breaks the *h* + *l* = 2*n* observation conditions for the *C*-centred cell, indicating a transformation to a new *P*2_1_/*n* cell (P 2) (Fig. 2 ▸).

**Figure 3:**
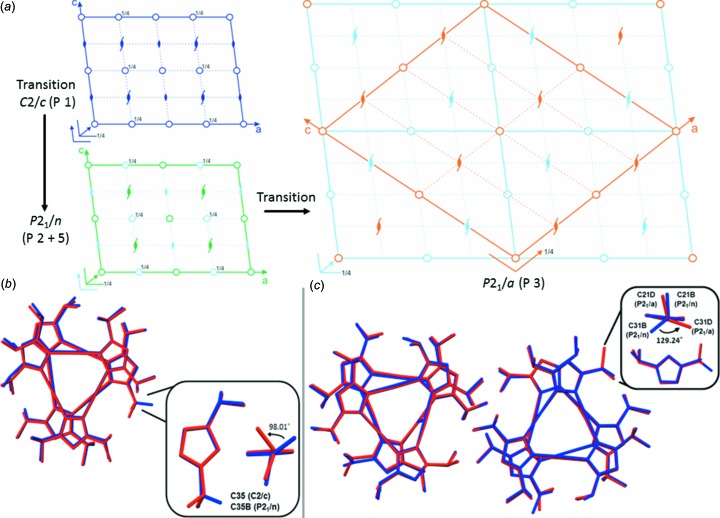
(*a*) Space-group diagrams displaying the symmetry of the mother *C*2/*c* (P 1) (top left), daughter *P*2_1_/*n* (P 2) (bottom left) and *P*2_1_/*a* (P 3) (right) phases of Form-I. The parts in light blue indicate symmetry elements that are no longer present in the new phases. (*b*) Overlay of the molecular skeleton of the complexes in the *C*2/*c* (P 1) (blue) and *P*2_1_/*n* (P 2) (red) cells, illustrating the molecular distortion which occurs during the phase change (inset). (*c*) Overlay of the molecular skeleton of the complexes in the *P*2_1_/*n* (1.69 GPa, blue) and *P*2_1_/*a* (2.18 GPa, red) phases to illustrate the molecular distortion which occurs during the phase change. Note the significant movement of the C31 atom through an angle of 129° between the *P*2_1_/*n* and *P*2_1_/*a* phases (inset).

The *P*2_1_/*n* phase is observed up to 2.03 GPa before a second phase transition is observed, again seen clearly in the *hl* planes of the diffraction pattern with the appearance of a second set of new Bragg peaks, this time at the position of 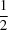 *h*, 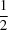 *l* of the mother cell, indicating a transition to a large *P*2_1_/*a* cell (demoted P 3), with double the unit-cell volume of the lower *P*2_1_/*n* phase (Fig. 3 ▸). Form-I remains in the *P*2_1_/*a* cell before transforming again between 2.43 and 2.70 GPa to a new larger *C*2/*c* cell (denoted P 4). Complete loss of the Bragg peaks associated with the previous *P*2_1_/*n* and *P*2_1_/*a* cells occurs and the pattern is replaced with intense reflections at ±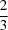 *h*, ±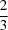 *l* (Fig. 3 ▸), indicating the formation of a new larger cell.

Finally, above 2.70 GPa, a fourth transition is observed to a *P*2_1_/*n* cell (denoted P 5). Visual inspection and indexing of the diffraction pattern confirms that the new phase is isostructural with the *P*2_1_/*n* phase observed at lower pressures, albeit a compressed form.

The re-entrant *P*2_1_/*n* phase may be identified clearly by the complete loss of the peaks associated with the large *C*2/*c* cell and the reappearance of the peaks at 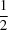 *h*, 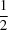 *l* associated with the primitive cells observed at lower pressures.

In order to understand the observed transitions and the reappearance of the re-entrant phase, analysis of the symmetry of the four phases and their relation to one another was performed. Structural refinement was also performed in order to understand the transitions in terms of the unit-cell contents and changes in molecular geometries. The transformation from the ambient *C*2/*c* (P 1) to *P*2_1_/*n* (P 2) phases can be easily rationalized as a loss of half the symmetry of the original *C*2/*c* mother cell (Fig. 3 ▸). The change in symmetry correlates to dramatic structural distortions within the unit cell, with a change in torsion angle in one of the iso­propyl groups by 98° (Fig. 3 ▸). The trend for loss of crystallographic symmetry continues with the transformation to the high-pressure *P*2_1_/*a* phase (P 4). The unit cell doubles in volume compared to the lower-pressure *C*2/*c* (P 1) and *P*2_1_/*n* (P 3) phases, once again correlating to significant structural distortions within the unit cell. In particular, the iso­propyl groups undergo significant re-arrangement, with one iso­propyl group rotating by 129.24° between the *P*2_1_/*n* and *P*2_1_/*a* phases (Fig. 3 ▸).

**Figure 4:**
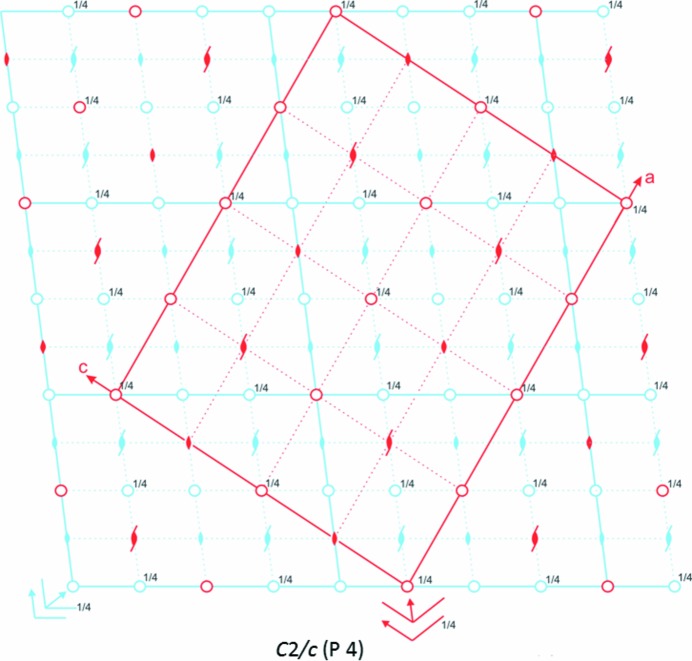
A space-group diagram displaying the symmetry of the high-pressure daughter *C*2/*c* phase (P 4) of Form-I. The part of the diagram corresponding to the original *C*2/*c* cell (P 1) is overlaid in light blue.

The transformation to the large *C*2/*c* cell (P 4) is harder to rationalize in terms of changes in crystallographic symmetry and distortions of molecular geometry, as the changes within the cell are more complex than those observed in the transitions between the other cells. Analysis of the symmetry involved shows that the daughter *C*2/*c* phase (P 4) is best described as a 3 × 3 supercell based on the ambient *C*2/*c* mother cell (P 1) with specific symmetry elements lost (Fig. 4 ▸). The appearance of the *C*2/*c* supercell breaks the previously observed trend of a progressive halving of unit-cell symmetry on transitioning between the *C*2/*c, P*2_1_/*n* and *P*2_1_/*a* cells (Fig. 2 ▸).

The transition to the large *C*2/*c* phase (P 4) is associated with a large structural distortion of some of the aurophilic interactions in the structure (0.25 and 0.27 Å for the Au1⋯Au2*B* and Au1*D*⋯Au2*C* distances, respectively), similar in size to that observed at low temperature by Yang *et al.* (2006 ▸), and is associated with a blue shift in the emission spectrum upon cooling.

In our high-pressure study, the low-pressure phases display a similar shortening of the Au⋯Au distances under com­pression; however, in the high-pressure *C*2/*c* phase, the distortion becomes asymmetric, with some interactions getting longer and others shorter (Table 1 ▸). The observed trends in the Au⋯Au distances can be corroborated independently with luminescence-band maxima, as was established in the study by Yang *et al.* (2006 ▸). One of the Au⋯Au distances in Form-I decreases by 0.24 Å (Au1⋯Au2*B* and Au1*D*⋯Au2*C*) between 2.18 and 2.7 GPa. The emission maximum decreases correspondingly by 450 cm^−1^ at 1800 cm^−1^/GPa, smaller by almost an order of magnitude than for bis­(di­ethyl­dithio­carbamato)gold(I). The difference is due to the initially longer Au⋯Au distances in Form-I compared to related complexes.

The luminescence spectra of Form-I under ambient conditions display a single band with a maximum at 13860 cm^−1^ and a full-width half-maximum height of 900 cm^−1^, similar in energy to Au(CN)_2_, but significantly narrower, by at least a factor of two. Peak widths of comparable size have been reported for di­thio­carbamate compounds, with Au⋯Au Au⋯Au antibonding HOMO (highest occupied molecular orbital) and bonding LUMO (lowest unoccupied molecular orbital) levels, with the low-frequency Au–Au modes, leading to a relatively narrow band (Baril-Robert *et al.*, 2012 ▸). For Au(CN)_2_^−^, decreasing distances in intermolecular aurophilic interactions have been shown to cause shifts to lower energy on the order of several thousand cm^−1^/GPa if the ambient-pressure distances are on the order of 3.0 to 3.4 Å.

**Figure 5:**
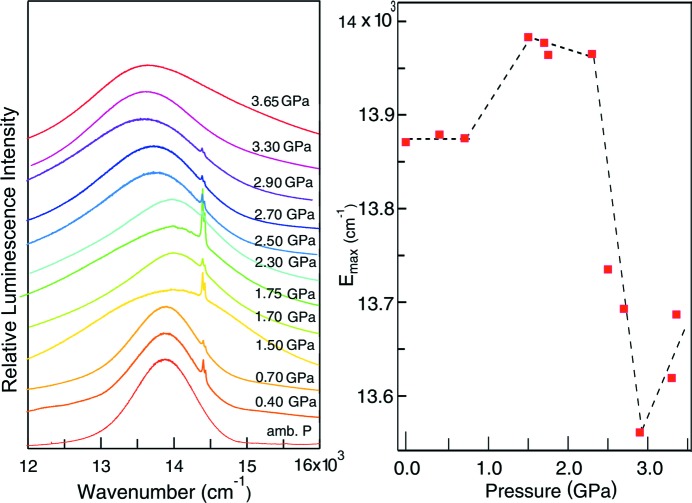
(Left) Luminescence spectra of Form-I at hydro­static pressures in the range 0.25–4.10 GPa (λ_ex_ = 488 nm). (Right) The corresponding position of the peak emission energy, *E*_max_, as a function of pressure.

In Form-I, the Au⋯Au distances are on the order of 3.3–3.4 Å at pressures below 1 GPa and the pressure-induced decrease of distance does not appear to influence the luminescence band maxima, leading to the constant values in this pressure range (Fig. 5 ▸). The trend changes dramatically at pressures above 2.2 GPa, with a strong decrease of the luminescence band maximum or red shift by −630±100 cm^−1^/GPa. The value is of the same order of magnitude as the shift of −1200 cm^−1^/GPa reported for bis­(di­ethyl­dithio­car­bam­ato)gold(I) (Baril-Robert *et al.*, 2012 ▸). This comparison is interesting, as the structure of this system has been reported at high pressure, with a decrease of the Au⋯Au distance by −0.1 Å/GPa (Paliwoda *et al.*, 2014 ▸), leading to a variation of 12000 cm^−1^/Å.

The reappearance of the *P*2_1_/*n* phase at 3.32 GPa makes the high-pressure behaviour of Form-I a rare example of a re-entrant phase transition. This can be confirmed through examination of the diffraction pattern and through direct com­parison of the refined structures, which both reveal the high-pressure *P*2_1_/*n* phase to be compressed but structurally identical to the low-pressure phase. There are a number of features that make this example unique in the literature, *viz.* that there are two intermediary phases observed and that pressure, rather than temperature, is the driving force.

### 3.2. Computational analysis   

To complement the high-pressure crystallographic and spectroscopic studies on Form-I, the effect of hydro­static pressure on the ambient *C*2/*c* and high-pressure *P*2_1_/*n* and *P*2_1_/*a* phases was modelled using density-functional theory (DFT). Unfortunately, due to the size of its unit cell, we were not able to model the larger high-pressure *C*2/*c* phase.

**Figure 6:**
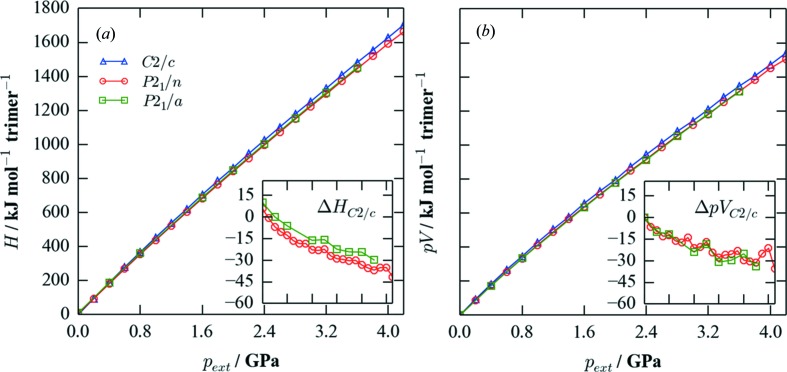
Calculated enthalpies, 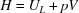, of the *C*2/*c, P*2_1_/*n* and *P*2_1_/*a* phases as a function of applied hydro­static pressure, 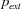, from 0 to 4.2 GPa. The values are shown relative to the enthalpy of the ambient *C*2/*c* phase at zero applied pressure. Part (*a*) shows *H*, while part (*b*) shows the contribution of the *pV* term on the same scale. In both plots, the inset figures show the differences in *H*/*pV* between the high-pressure phases and the *C*2/*c* phase as a function of pressure, over the same pressure range as the parent plot.

**Figure 7:**
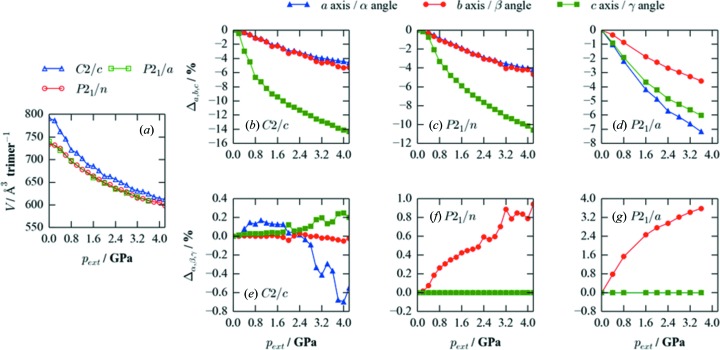
(*a*) Optimized cell volume and percentage changes in lattice parameters of (*b*)/(*e*) the *C*2/*c*, (*c*)/(*f*) the *P*2_1_/*n* and (*d*)/(*g*) the *P*2_1_/*a* phases as a function of applied hydro­static pressure, 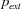, from 0 to 4.2 GPa.

Taking the unit cells obtained from the refinement of the ambient and high-pressure diffraction patterns as a starting point, the cell shape, volume and internal positions were optimized to a tolerance of 10^−3^ eV under applied hydro­static pressures from 0.0 to 4.2 GPa in steps of 0.2 and 0.4 GPa for the *C*2/*c* and *P*2_1_/*n*, and *P*2_1_/*a* phases, respectively. We note that this tolerance is approximately an order of magnitude tighter than the calculated enthalpy differences between the phases. Fig. 6 ▸ shows the calculated enthalpies, 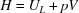, where 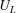 is the lattice internal energy, of the three phases as a function of pressure, relative to the enthalpy of the ambient *C*2/*c* phase at zero pressure. Application of pressure leads to a substantial increase in energy, with the enthalpies at 4.2 GPa being approximately 1700 kJ mol^−1^ per trimer higher than at zero applied pressure. A separate plot of the *pV* term shows that this, rather than differences in the (lattice) internal energy, 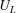, is the main contributor to the increase in enthalpy. However, whereas the absolute energies vary significantly, the per-trimer energy differences between the three forms (inset plots) are much smaller, on the order of a few tens of kJ mol^−1^. These calculations suggest the low-pressure *C*2/*c* phase to be the most stable at ambient pressure, while a small applied hydro­static pressure is sufficient to favour both the high-pressure *P*2_1_/*n* and *P*2_1_/*a* phases. This is largely due to the fact that the *P*2_1_/*n* and *P*2_1_/*a* phases both have a higher predicted density than the *C*2/*c* phase (Fig. 7 ▸), leading to a smaller *pV* contribution to the high-pressure enthalpy (Fig. 6 ▸). The calculated enthalpies predict that the two high-pressure phases are energetically close, but do not display the crossing expected to occur at around 2 GPa. Moreover, the calculations also predict that the *C*2/*c* and *P*2_1_/*n* phases should be in equilibrium at around 0.2 GPa, which is considerably lower than the pressure of 1 GPa at which the phase transition is observed by diffraction.

One possible origin for this discrepancy is that the energetics are sensitive to weak intramolecular interactions (*e.g*. van der Waals forces), the subtleties of which are not accurately captured by the PBEsol functional.

To probe the changes in geometry that occur to the three phases under compression, we compared the optimized cell volume and lattice parameters as a function of pressure (Fig. 7 ▸). According to these calculations, the ambient-pressure *C*2/*c* phase consistently has the largest volume per trimer over the range of pressures explored – although the difference narrows at higher pressures – whereas the *P*2_1_/*n* and *P*2_1_/*a* phases have similar densities, both being higher than that of the *C*2/*c* phase. As noted above, this appears to be the main origin of the *P*2_1_/*n* and *P*2_1_/*a* phases becoming energetically favoured under applied pressure.

Interestingly, all three phases display a differential com­pression of the three crystallographic axes under pressure, with the *c* axis shrinking more than the *a* and *b* axes in the *C*2/*c* and *P*2_1_/*n* phases, and the *a* and *c* axes compressing more the than the *b* axis in the *P*2_1_/*a* phase. Structurally, this corresponds to a reduction in the spacing between the layers of hexameric units, indicating that the steric interaction between layers is weaker (*i.e*. less unfavourable) than that between the molecular units within a layer. This observation explains the large structural distortions to the iso­propyl groups under pressure (Fig. 3 ▸), since the methyl groups can project into the interlayer space, and would thus need to rearrange as this was compressed. As with the axis lengths, the cell angles likewise exhibit differential changes under pressure, which are again consistent with a compression of the spacing between layers.

**Figure 8:**
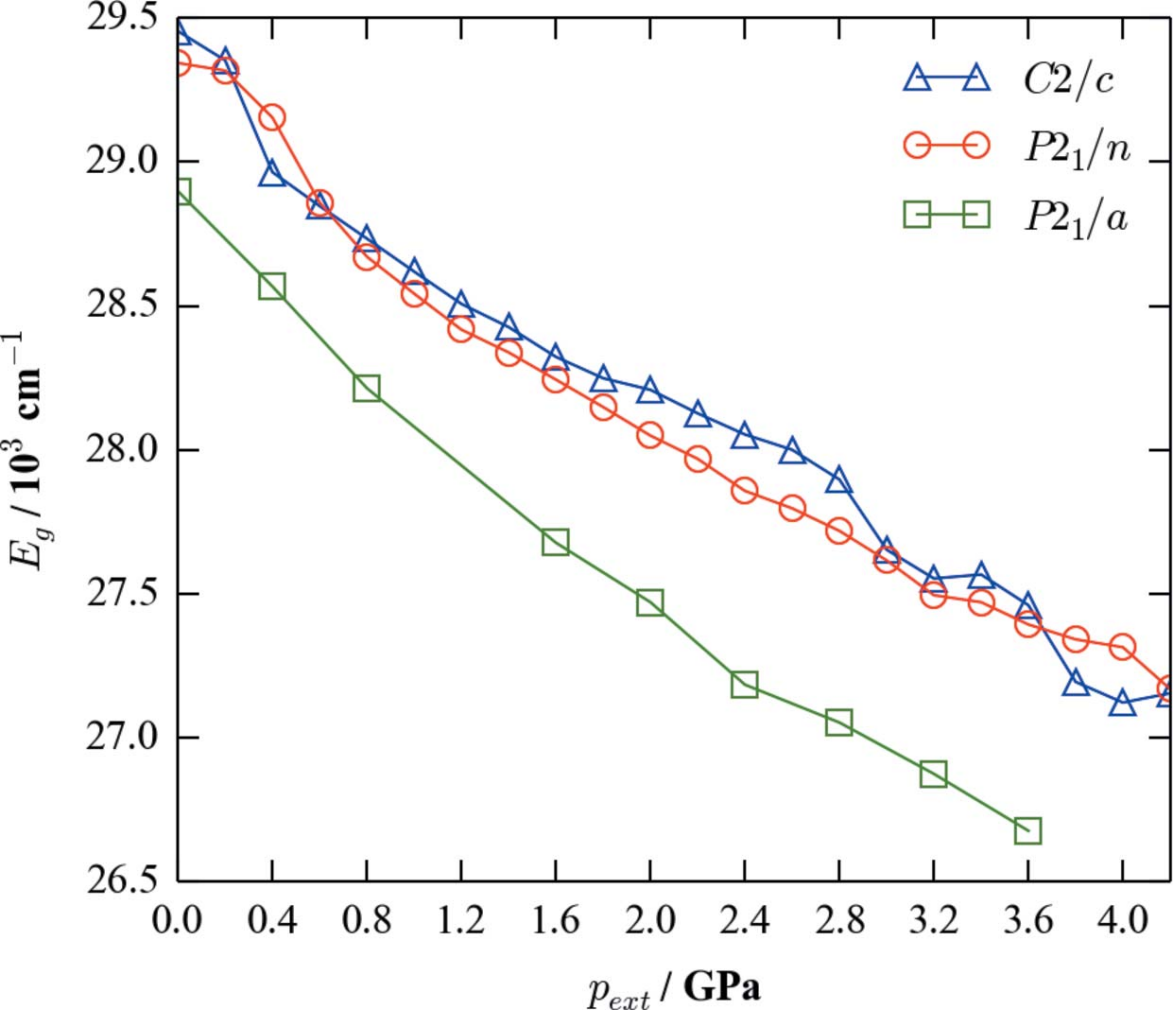
Energy gap between the highest-occupied and lowest-unoccupied Kohn–Sham bands in the *C*2/*c, P*2_1_/*n* and *P*2_1_/*a* phases as a function of applied hydro­static pressure, 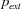, from 0 to 4.2 GPa.

Finally, to confirm whether the experimentally observed shift in luminescence is due to changes in the HOMO–LUMO gap with pressure, we extracted the difference between the highest-occupied and lowest-unoccupied Kohn–Sham bands in our optimized structures (Fig. 8 ▸). This analysis suggests that the bandgap of all three phases would decrease significantly under pressure; this is the reverse trend to what would be anticipated from a simple covalent-bonding picture, in which as atoms approach more closely the increased orbital overlap should lead to a larger separation between bonding and antibonding states. It is thus reasonable to infer that the change in the energy gap is dictated more by changes in conformation than by simple compression of interatomic distances. Interestingly, as a qualitative trend, the *P*2_1_/*a* phase consistently has a smaller gap than the (ambient) *C*2/*c* and *P*2_1_/*n* phases, which is consistent with a red shift in the luminescence. This suggests that the transition to the *P*2_1_/*a* phase, as well as presumably the transition to the large *C*2/*c* phase at higher pressures, may be responsible for the red shift in the luminescence evident in Fig. 8 ▸.

We note that PBEsol is not expected to give accurate energy gaps and, indeed, that the electronic gap in the ground state is unlikely to correspond to the maximum in the emission spectra (Skelton *et al.*, 2015 ▸). This is due to the effects of electronic and/or ionic relaxation in the excited state, neither of which are represented in the calculation of the LUMO levels in a standard DFT calculation, plus the possibility of many-body effects which are likewise not well described by standard generalized-gradient approximation (GGA) DFT functionals. However, as with the lattice dynamics, the higher levels of theory needed to accurately describe these effects would be prohibitively expensive for the large unit cells being modelled in the present work.

Despite these caveats, the calculations do illustrate both that the HOMO–LUMO gap is sensitive to pressure and that the significant changes in geometry which occur through the phase transition may account for the correspondingly dramatic changes in the emission profile.

### 3.3. Comparative structural studies of Form-II   

Our discovery of the Form-II polytype provides the opportunity to investigate whether the complex crystallographic behaviour of Form-I is a product of the increased steric interaction observed between iso­propyl groups aggregated together through the aurophilic interactions or whether it can be understood in terms of the differences in molecular stacking arrangement, *i.e.* interactions between the layers of stacked trimers.

**Figure 9:**
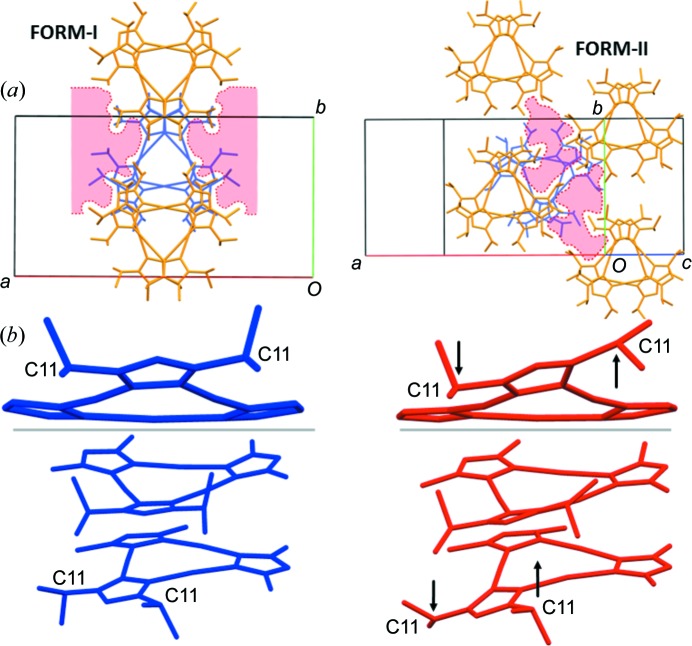
(*a*) Packing diagrams showing the molecular overlap between one layer (blue) and the layer above (orange) in Form-I (left) and Form-II (right). Shaded areas indicate those which are not near the iso­propyl groups of the molecules, and thus areas where atoms are likely to have the most freedom to change position. (*b*) Comparison of the ambient and 2.88 GPa structures of Form-II. Note the distortion of the triazole ring and the associated iso­propyl groups on the closest triazole.

Single-crystal diffraction revealed that Form-II does not display the same complex phase behaviour as Form-I, remaining in the same *C*2/*c* phase from ambient to 2.88 GPa. However, the molecules do undergo significant structural distortion with increased pressure. In particular, the five-membered triazole rings twist out of the plane relative to the gold-based trimer to accommodate the increase in pressure (Fig. 9 ▸).

The differing behaviour of the two forms under pressure, despite having near-identical molecular geometries, confirms that the dramatic changes observed in Form-I are likely due to a combination of competing processes, *viz.* minimizing unfavourable steric interactions between the iso­propyl groups on the aurophilically bound trimers and reducing the volume per molecule to minimize enthalpy. In Form-I, the trimers stack almost directly upon one another and are, as a result, sterically congested, making it impossible to reduce the volume without worsening energetically unfavourable steric interactions with the molecules stacked above and below in the unit cell. Conversely, in Form-II, the layers of molecules have a larger offset with respect to one another. The efficient packing at room temperature, enables the structure to accommodate compression through continuous structural distortion rather than the sudden conformational changes that result in phase transitions for Form-I.

## 4. Conclusions   

The high-pressure crystallography reported here demonstrates that molecular crystals are equally capable of displaying phase behaviour as complex as simpler elemental materials and, indeed, in the case of Form-I, a more complex phase behaviour involving four single-crystal-to-single-crystal phase transitions to form a re-entrant phase has been observed. Such behaviour is perhaps more accessible due to the lower hydro­static pressures required. The technique of using unusual intermolecular interactions to bring sterically demanding groups into closer proximity than in typical packing arrangements in order to orchestrate complex phase behaviour is a technique that has potential and should be explored further, designing systems with predictable phase behaviour. The structural investigation of the two polytypes of tris(μ_2_-3,5-diiso­propyl-1,2,4-triazolato-κ^2^ *N* ^1^:*N* ^2^)trigold(I) further demonstrates the role that packing effects can have on the phase behaviour of molecular crystals, while high-pressure luminescence spectroscopy corroborates previous conclusions regarding the susceptibility of aurophilic interactions to the effects of high hydro­static pressure.

## Supplementary Material

Crystal structure: contains datablock(s) 0.00GPa_296K_Au3triazole-FORM-I, 0.21GPa_Au3triazole-FORM-I, 0.41GPa_Au3triazole-FORM-I, 0.66GPaAu3triazole-FORM-I, 0.97GPa_Au3triazole-FORM-I, 1.69GPa_Au3triazole-FORM-I, 2.18GPa_Au3triazole-FORM-I, 2.70GPa_Au3triazole-FORM-I, 3.31GPaAu3triazole-FORM-I, 0.00GPa_296K_Au3triazole-FORM-II, 0.63GPa_Au3triazole-FORM-II, 1.07GPa_Au3triazole-FORM-II, 1.25GPa_Au3triazole-FORM-II, 1.93GPa_Au3triazole-FORM-II, 2.26GPa_Au3triazole-FORM-II, 2.51GPa_Au3triazole-FORM-II, 2.88GPa_Au3triazolep-FORM-II. DOI: 10.1107/S2052252516013129/ed5009sup1.cif

Pictures of the crystals and the diffraction patterns under the range of pressures, crystallographic tables, etc.. DOI: 10.1107/S2052252516013129/ed5009sup2.pdf

Structure factors: contains datablock(s) shelx. DOI: 10.1107/S2052252516013129/ed50090.00GPa_296K_Au3triazole-FORM-Isup3.hkl

Structure factors: contains datablock(s) shelx. DOI: 10.1107/S2052252516013129/ed50090.21GPa_Au3triazole-FORM-Isup4.hkl

Structure factors: contains datablock(s) shelx. DOI: 10.1107/S2052252516013129/ed50090.41GPa_Au3triazole-FORM-Isup5.hkl

Structure factors: contains datablock(s) shelx. DOI: 10.1107/S2052252516013129/ed50090.66GPaAu3triazole-FORM-Isup6.hkl

Structure factors: contains datablock(s) shelx. DOI: 10.1107/S2052252516013129/ed50090.97GPa_Au3triazole-FORM-Isup7.hkl

Structure factors: contains datablock(s) new1. DOI: 10.1107/S2052252516013129/ed50091.69GPa_Au3triazole-FORM-Isup8.hkl

Structure factors: contains datablock(s) shelx. DOI: 10.1107/S2052252516013129/ed50092.18GPa_Au3triazole-FORM-Isup9.hkl

Structure factors: contains datablock(s) shelxl. DOI: 10.1107/S2052252516013129/ed50093.31GPaAu3triazole-FORM-Isup10.hkl

Structure factors: contains datablock(s) shelx. DOI: 10.1107/S2052252516013129/ed50090.00GPa_296K_Au3triazole-FORM-IIsup11.hkl

Structure factors: contains datablock(s) shelx. DOI: 10.1107/S2052252516013129/ed50090.63GPa_Au3triazole-FORM-IIsup12.hkl

Structure factors: contains datablock(s) shelx. DOI: 10.1107/S2052252516013129/ed50091.07GPa_Au3triazole-FORM-IIsup13.hkl

Structure factors: contains datablock(s) shelx. DOI: 10.1107/S2052252516013129/ed50091.25GPa_Au3triazole-FORM-IIsup14.hkl

Structure factors: contains datablock(s) shelx. DOI: 10.1107/S2052252516013129/ed50091.93GPa_Au3triazole-FORM-IIsup15.hkl

Structure factors: contains datablock(s) shelx. DOI: 10.1107/S2052252516013129/ed50092.26GPa_Au3triazole-FORM-IIsup16.hkl

Structure factors: contains datablock(s) shelx. DOI: 10.1107/S2052252516013129/ed50092.51GPa_Au3triazole-FORM-IIsup17.hkl

Structure factors: contains datablock(s) shelxl. DOI: 10.1107/S2052252516013129/ed50092.70GPa_Au3triazole-FORM-Isup18.hkl

Structure factors: contains datablock(s) shelx. DOI: 10.1107/S2052252516013129/ed50092.88GPa_Au3triazolep-FORM-IIsup19.hkl

CCDC references: 1405869, 1405870, 1405871, 1405872, 1405873, 1405874, 1405875, 1405876, 1405877, 1405878, 1405879, 1405880, 1405881, 1405882, 1405883, 1405884, 1405868

## Figures and Tables

**Table 1:** Aurophillic interaction lengths of Form-I

		Distance (Å)						
GPa	Phase	Inter-trimer (Molecule 1)			Inter-trimer (Molecule 2)		
Pressure		Au1—Au2*B*	Au1*B*—Au2	Au3—Au3*B*	Au1*C*—Au2*D*	Au1*D*—Au2*C*	Au3*C*—Au3*D*
0.00	*C*2/*c*	3.4070 (5)	3.4070 (5)	3.4604 (4)	–	–	–
0.21	*C*2/*c*	3.3653 (11)	3.3653 (11)	3.4287 (14)	–	–	–
0.41	*C*2/*c*	3.3028 (13)	3.3028 (13)	3.3851 (15)	–	–	–
0.66	*C*2/*c*	3.2599 (12)	3.2599 (12)	3.3909 (15)	–	–	–
0.97	*C*2/*c*	3.2309 (110)	3.2309 (10)	3.3784 (12)	–	–	–
1.69	*P*2_1_/*n*	3.2105 (12)	3.1192 (12)	3.3573 (13)	–	–	–
2.18	*P*2_1_/*a*	3.2990 (30)	3.063 (30)	3.3490 (30)	3.1600 (30)	3.3320 (30)	3.3320 (30)
2.70	*C*2/*c*	3.0592 (15)	3.0592 (15)	3.2440 (10)	3.2525 (11)	3.0531 (10)	3.3501 (12)
3.31	*P*2_1_/*n*	3.1526 (16)	3.0273 (15)	3.2866 (19)	–	–	–
