# Supplementary material for: Observation of a re-entrant phase transition in the molecular complex tris(μ2-3,5-diiso­propyl-1,2,4-triazolato-κ2 N 1:N 2)trigold(I) under high pressure
Source: IUCrJ. 2016 Aug 18;3(Pt 5):367–76. doi: 10.1107/S2052252516013129 (PMC5391858; doi:10.1107/S2052252516013129)
Supplement: Supplementary file 2 [file m-03-00367-sup2.pdf]

IUCrJ

Volume 3 (2016)

Supporting information for article:

Observation of a re-entrant phase transition in the molecular complex tris(μ2-3,5-diisopropyl-1,2,4-triazolato-κ^{2}N^{1}:N^{2})trigold(I) under high pressure

Christopher H. Woodall, Jeppe Christensen, Jonathan M. Skelton, Lauren E. Hatcher, Andrew Parlett, Paul R. Raithby, Aron Walsh, Stephen C. Parker, Christine M. Beavers, Simon J. Teat, Mourad Intissar, Christian Reber and David R. Allan

Supporting information

S1. Contents

Crystal fracturing photos 2

Axial diffraction Images from the (*h*0*l*) of Form-I 4

Relative Bragg Peak intensities from the (*h*1*l*) layer of Form-I 5

High-pressure Raman Spectra for Form-I 7

Crystallographic data of *tris*(μ_2-3,5-diisopropyl-1,2,4-triazolato-*N,N'*)-tri-gold(I) 8

S2. Crystal Fracturing Photos

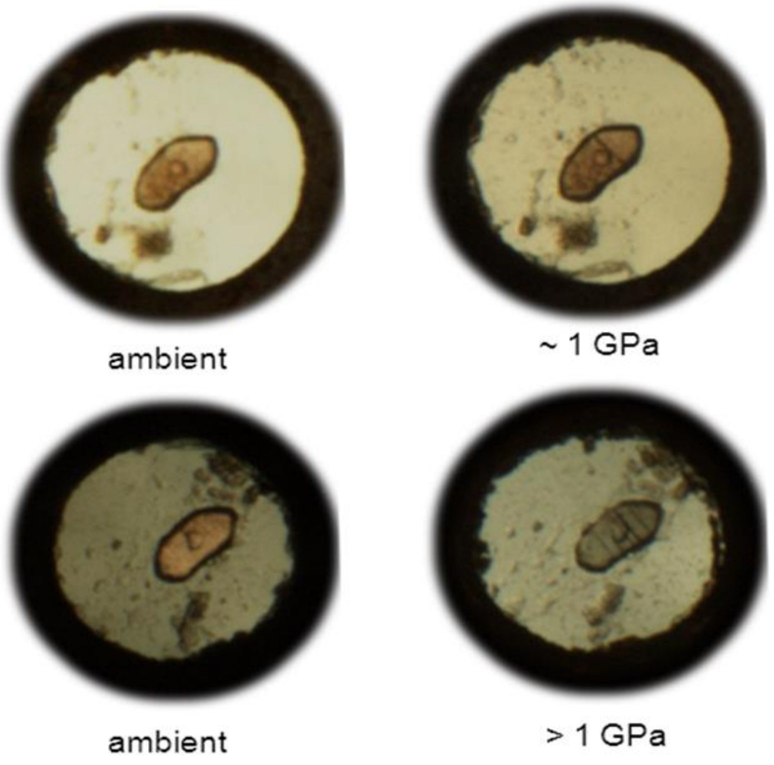

Figure S1 . Crystal of Form-1 of *tris*(μ_2-3,5-diisopropyl-1,2,4-triazolato-*N,N'*)-tri-gold(I). Note the lateral crack that appears in the ~ 1 GPa image, disappears in the ambient and reappears along with multiple other cracks in the > 1 GPa image. The cracks permeate approximately along the [001] face of the crystal.

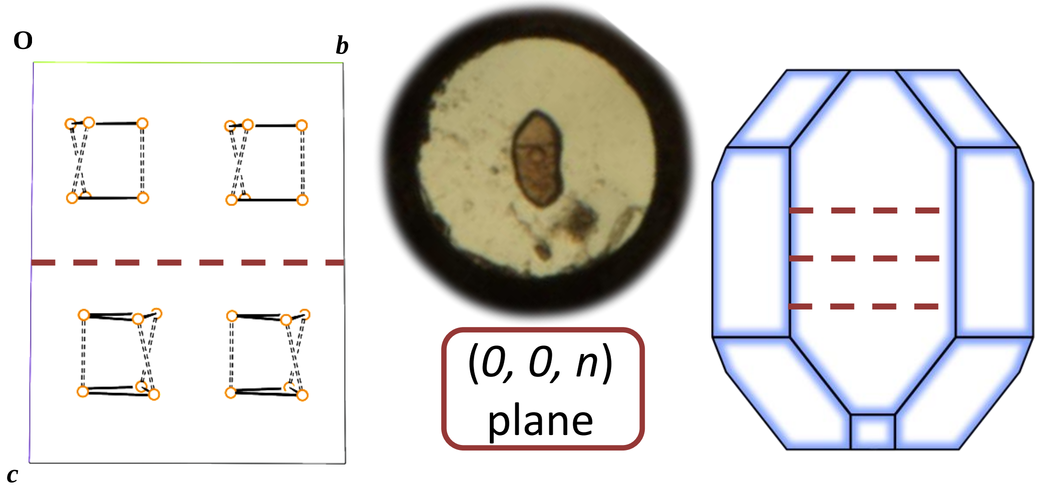

Left) Packing of Form-I viewed down the *a* axis, all atoms except gold omitted for clarity Centre) Close up of a single crystal of Form-I viewed in a DAC. Note the line permeating across the face. Right) Mercury generated crystal morphology of the 0.00 GPa structure of Form-I. The dashed crimson line indicates the plane of the crystal crack in the single crystal, as determined by face indexing in both the unit cell contents and generated crystal morphology.

S3. Axial diffraction Images from the (*h*0*l*) of Form-I

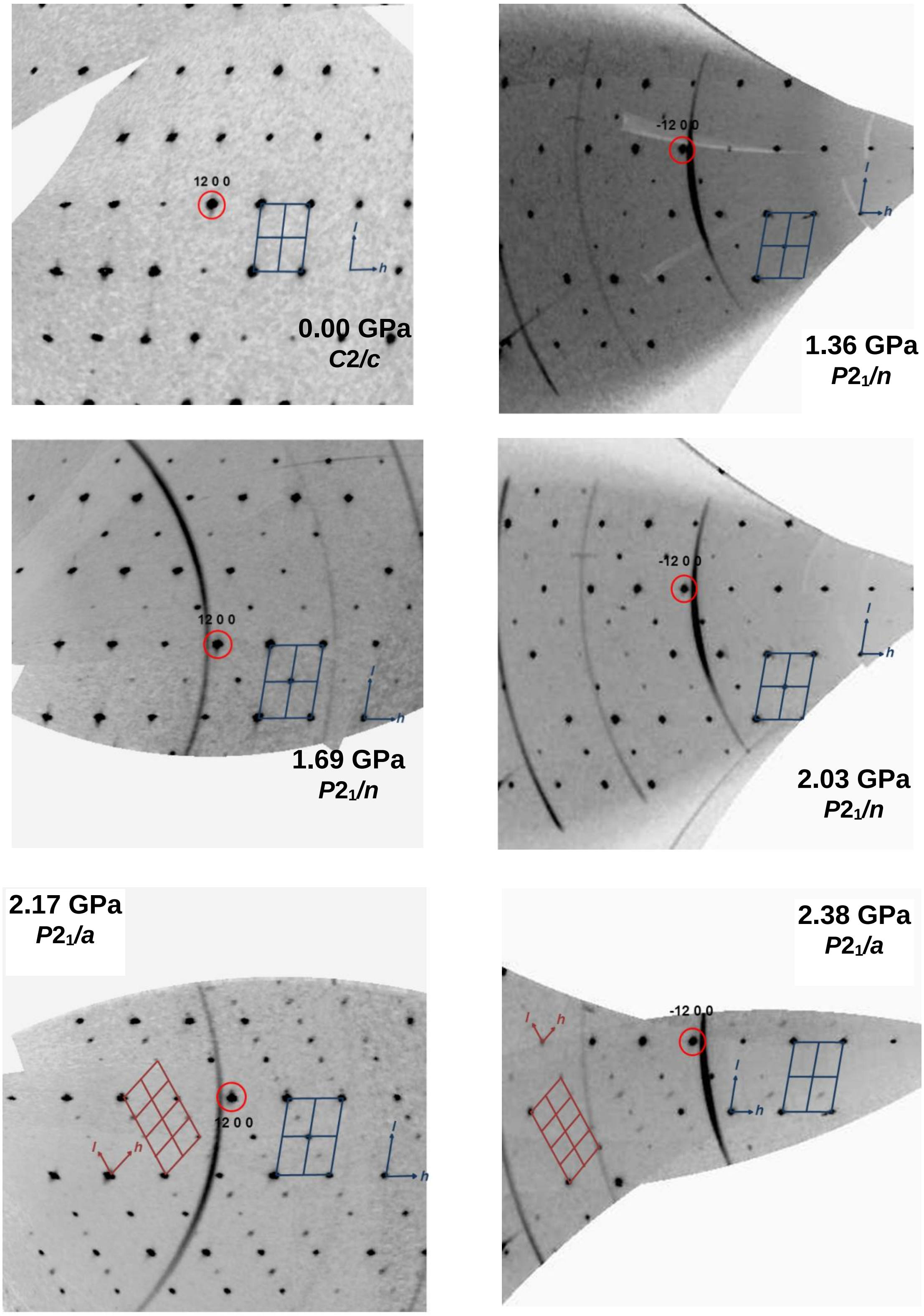

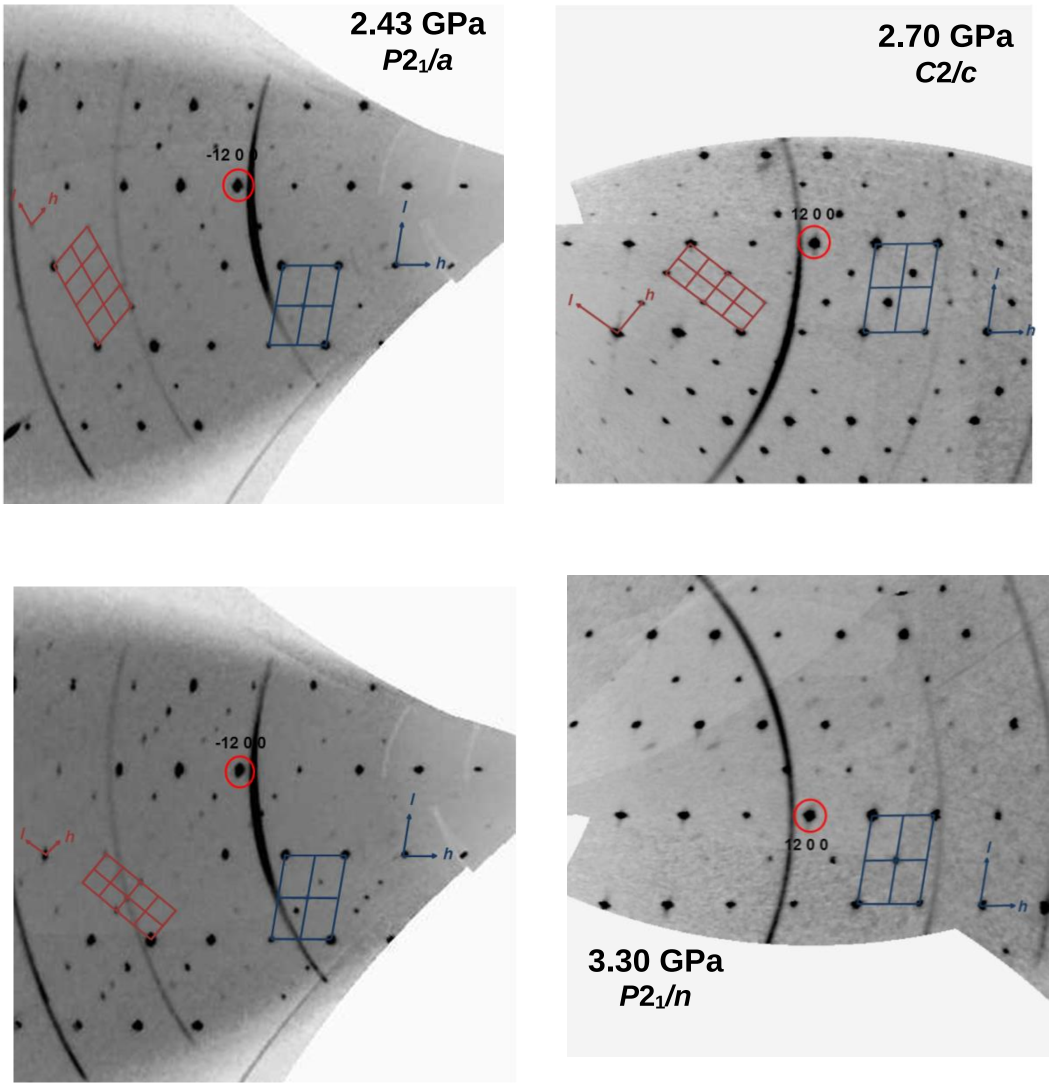

Figure S2 . (h0l) axial precession images of Form-I. The images have the 12 0 0 or -12 0 0 reflection of the mother C2/c phase circled as a reference reflection. The blue axes displayed reference to those of the C2/c mother phase while those in maroon represent the new high-pressure daughter phase.

S4. Relative Bragg Peak intensities from the (*h*1*l*) layer of Form-I

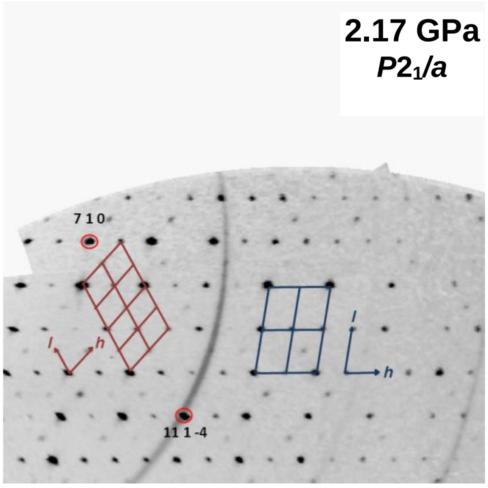

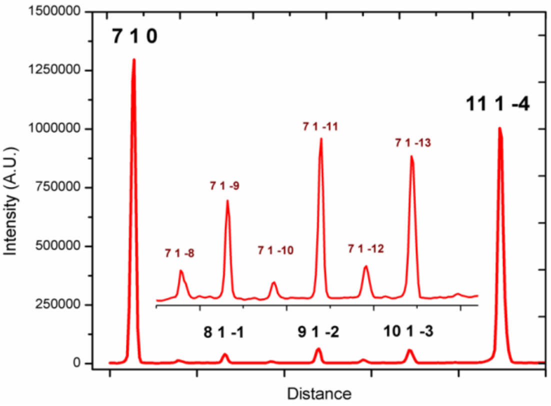

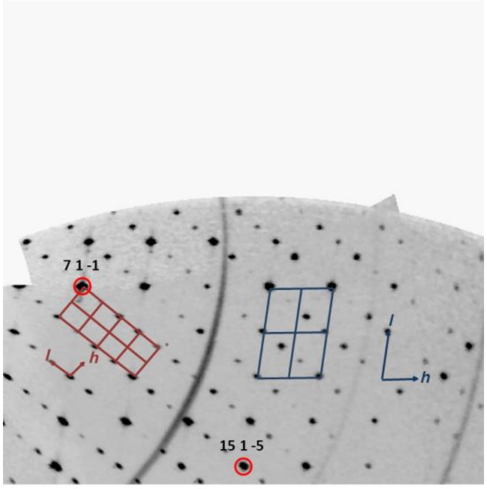

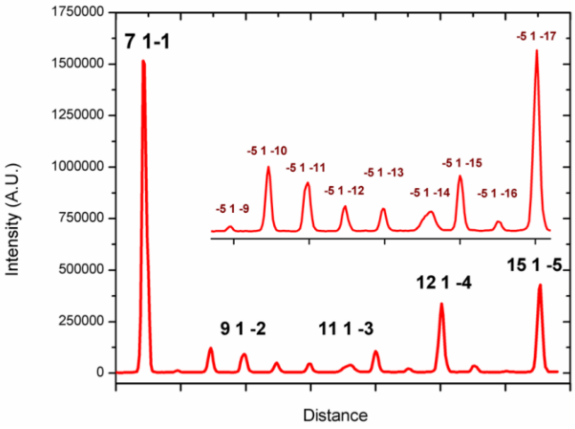

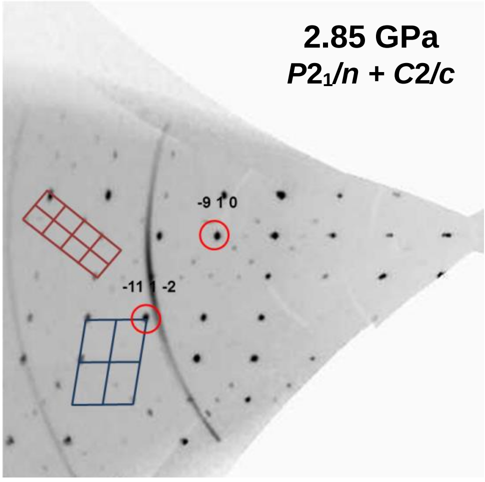

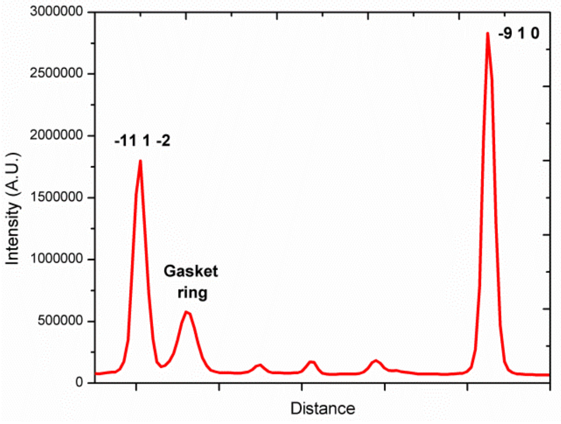

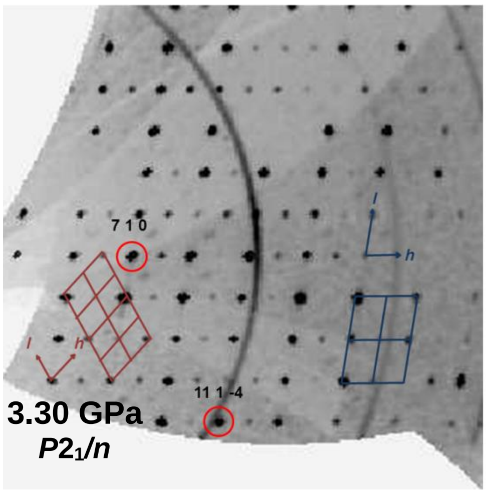

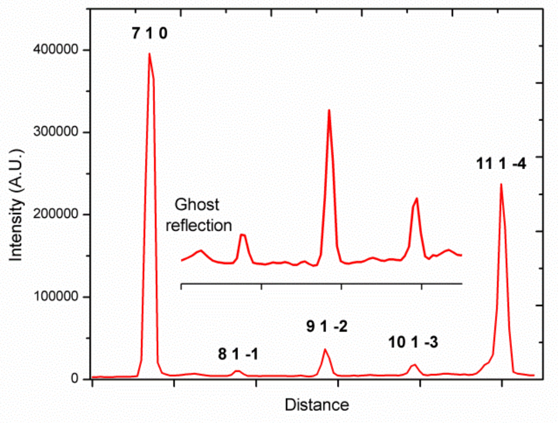

.

Figure S3 . Left) ($h1l$) axial precession images of Form-I, The blue axes displayed reference to those of the $C2/c$ mother phase while those in maroon represent the new high-pressure daughter phase. Right) Relative peak intensities taken along the line between the two reflections marked with red circles

S5. High Pressure Raman for Form I

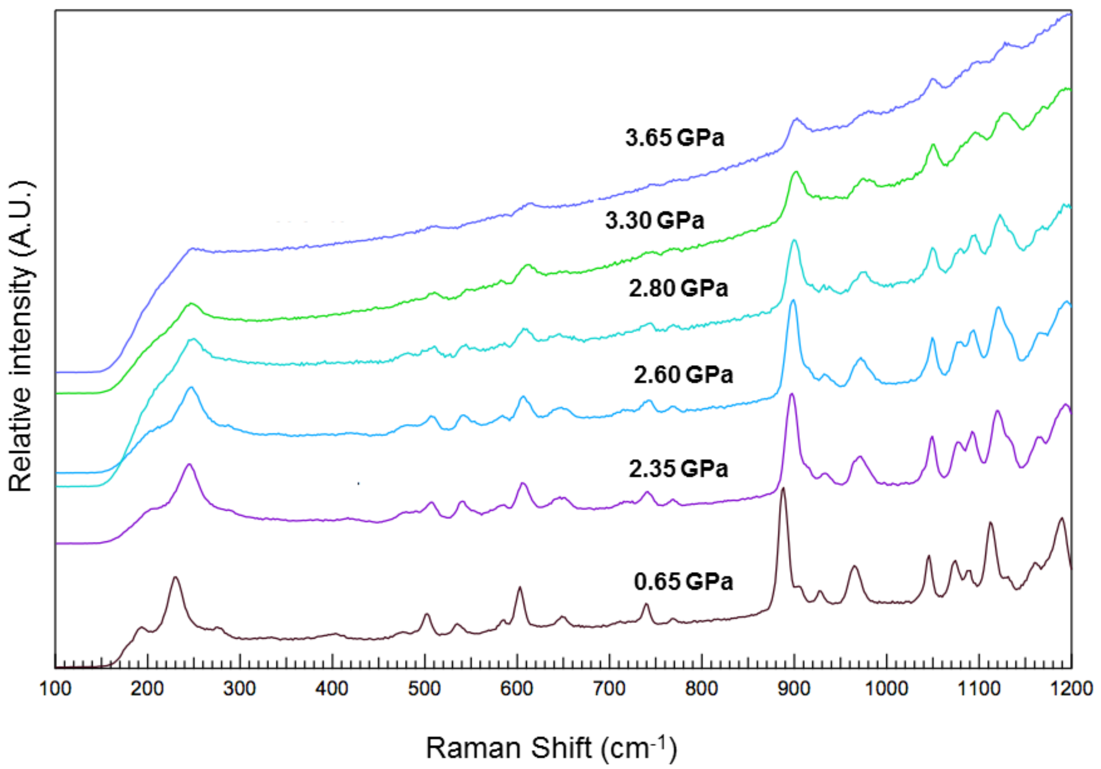

Figure S4 Raman spectrum

Table S1 Crystallographic data of *tris*(μ_2-3,5-diisopropyl-1,2,4-triazolato-*N,N'*)-tri-gold(I) at pressure (Form-I)

**Pressure (GPa)**	**ambient**	**0.21**	**0.41**	**0.66**	**0.97**	**1.69**	**2.18**	**2.70**	**3.31**
**Formula**	$C_{24}H_{42}N_9Au_3$	$C_{24}H_{42}N_9Au_3$	$C_{24}H_{42}N_9Au_3$	$C_{24}H_{42}N_9Au_3$	$C_{24}H_{42}N_9Au$	$C_{24}H_{42}N_9$Au3	$C_{24}H_{42}N_9$Au3	$C_{24}H_{42}N_9$Au3	$C_{24}H_{42}N_9$Au3
**M_r**	1047.57	1047.57	1047.57	1047.57	1047.57	1047.57	1047.57	1047.57	1047.57
**Crystal System**	monoclinic	monoclinic	monoclinic	monoclinic	monoclinic	Monoclinic	monoclinic	monoclinic	monoclinic
**Space Group**	*C 2/c*	*C 2/c*	*C 2/c*	*C 2/c*	*C 2/c*	$P2_1/n$	$P2_1/a$	*C2/c*	$P2_1/n$
**a,b,c (Å)**	25.058(5)<br>14.038(3)<br>18.102(4)	24.7872(11)<br>13.8653(8)<br>17.7857(13)	24.641(4)<br>13.7018(6)<br>17.5317(9)	24.290(5)<br>13.584(3)<br>17.379(4)	24.215(5)<br>13.516(3)<br>17.266(4)	23.765(5)<br>13.396(3)<br>16.789(3)	26.529(5)<br>13.396(3)<br>31.094(6)	37.163(7)<br>13.417(3)<br>30.421(6)	23.3975(14)<br>13.3010(11)<br>16.1373(15)
**ß (°)**	97.313(3)	97.493(4)	97.572(8)	98.51(3)	98.70(3)	99.58(3)	110.11(3)	88.48(3)	99.901(6)
**V (Å^3)**	6316(2)	6060.4(6)	5867.6(9)	5671(2)	5586.0(21)	5270.2(18)	10376(4)	15163(5)	4947.3(7)
**Z/Z'**	8/1	8/1	8/1	8/1	8/1	8/2	16/4	24/3	4/1
**D_x (Mg m^{-3})**	2.203	2.296	2.372	2.454	2.491	2.641	2.682	2.753	2.813
**μ(mm^{-1})**	11.555	12.042	12.438	12.869	13.065	13.848	14.067	14.439	14.752
**F(000)**	3888	3888	3888	3888	3888	3888	7776	11664	3888
**T_{min} /T_{max}**	0.7933/1.0000	0.7903/1.0000	0.6227/1.0000	0.8456/0.9911	0.7829/1.0000	0.8094/0.9707	0.7677/ 0.9565	0.8074/0.9730	0.8620/ 1.0000
**2θ range(°)**	2.79/28.60	3.13/28.60	2.65/28.60	1.62/28.59	1.63/26.53	1.84/28.69	1.28/ 28.60	1.26/31.07	1.58/ 24.76
**Rint**	0.0668	0.0548	0.0732	NA	NA	NA	NA	NA	0.0777
**Completeness**	0.997	0.465	0.307	0.315	0.347	0.524	0.458	0.490	0.570
**No. Measured/ Independent Observed reflections**	42239/9621	19816/4311	17499/2746	2725/2725	2409/2409	8498/8498	14516/14516	14228/14228	22064/ 5768
**R[F]/wR[F2] (for I >2s)**<br>**R[F]/wR[F2] (all data)**	0.0392/0.1088<br>0.0577/0.1215	0.0557/0.1940<br>0.0741/0.2302	0.0549/0.1573<br>0.0758/0.1891	0.0466/0.1342<br>0.0712/0.1748	0.0408/0.1260<br>0.0484/0.1336	0.0564/0.1650<br>0.0830/0.2026	0.1321/0.2568<br>0.1853/0.2903	0.0495/0.1434<br>0.0720/0.1723	0.0693/ 0.1940<br>0.1036/ 0.2246
**Restraints/Parameters**	0 /360	68/166	68/163	68/166	68/165	114/342	239/653	170/514	116/332
**$\Delta\rho_{max}$ $\Delta\rho_{min}$(e Å^{-3})**	0.982/-1.014	1.708/-1.289	1.124/-1.548	1.100/-1.268	0.940/-1.039	2.176/-1.824	8.165/-7.292	3.457/-2.747	1.786/ -1.606

Table S2 Crystallographic data of *tris*(μ_2-3,5-diisopropyl-1,2,4-triazolato-*N,N'*)-tri-gold(I) at pressure (Form-II)

Pressure (GPa)	0.00	0.63	1.07	1.25	1.93	2.26	2.51	2.88
Formula	$C_{24}H_{42}N_9Au_3$	$C_{24}H_{42}N_9Au_3$	$C_{24}H_{42}N_9Au_3$	$C_{24}H_{42}N_9Au_3$	$C_{24}H_{42}N_9Au$	$C_{24}H_{42}N_9Au$	$C_{24}H_{42}N_9Au$	$C_{24}H_{42}N_9Au$
M_r	1047.57	1047.57	1047.57	1047.57	1047.57	1047.57	1047.57	1047.57
Crystal System	monoclinic	monoclinic	monoclinic	monoclinic	monoclinic	monoclinic	monoclinic	monoclinic
Space Group	*C 2/c*	*C 2/c*	*C 2/c*	*C 2/c*	*C 2/c*	*C 2/c*	*C 2/c*	*C 2/c*
a,b,c (Å)	24.316(4)<br>14.341(2)<br>20.951(5)	23.7794(13)<br>14.0552(6)<br>20.087(2)	23.6575(13)<br>13.9552(6)<br>19.838(4)	23.5723(13)<br>13.8758(6)<br>19.647(4)	23.4383(14)<br>13.7533(6)<br>19.341(4)	23.4449(14)<br>13.6963(7)<br>19.285(2)	23.3640(12)<br>13.6258(7)<br>19.112(2)	23.3195(10)<br>13.5632(7)<br>18.951(3)
ß (°)	121.105(2)	121.333(10)	121.445(6)	121.589(6)	121.812(6)	121.920(10)	122.080(9)	122.188(7)
V ($Å^3$)	6255(2)	5734.5(8)	5587.5(12)	5474.0(12)	5298.1(12)	5256.2(8)	5155.4(7)	5072.8(8)
Z/Z'	8/1	8/1	8/1	8/1	8/1	8/1	8/1	8/1
D_x (Mg m^{-3})	2.225	2.427	2.491	2.542	2.627	2.648	2.697	2.743
μ(mm^{-1})	11.667	13.676	14.036	14.327	14.803	14.921	15.212	15.460
F(000)	3888	3888	3888	3888	3888	3888	3888	3888
T_{min}/T_{max}	0.4384/0.7462	0.7571/1.0000	0.7755/1.0000	0.7862/1.0000	0.8029/1.0000	0.7740/1.0000	0.7629/1.000	0.7862/1.000
2θ range(°)	2.75/24.76	2.81/26.95	2.90/26.21	2.85/26.21	2.93/26.95	2.93/26.48	2.94/25.48	2.95/26.94
Rint	0.0552	0.1071	0.0389	0.0430	0.0385	0.0430	0.0433	0.0406
Completeness	0.995	0.374	0.377	0.387	0.378	0.321	0.407	0.385
No. Measured/ Independent Observed reflections	27525/6374	10652/2562	10005/2323	9926/2345	9760/2385	9831/2496	8906/2136	9203/2325
R[F]/wR[F2] (for I >2s)<br>R[F]/wR[F2] (all data)	0.0346/0.0895<br>0.0438/0.0949	0.0481/0.1248<br>0.0744/ 0.1542	0.0383/0.1004<br>0.0622/0.1204	0.0432/0.1146<br>0.0652/ 0.1362	0.0519/0.1354<br>0.0730/0.1540	0.0672/0.1703<br>0.1071/0.2299	0.0540/0.1209<br>0.0783/0.1405	0.0611/0.1463<br>0.0996/0.2107
Restraints/Parameters	41/337	33/164	33/166	33/165	27/165	27/163	27/163	29/163
$\Delta\rho_{max}$ $\Delta\rho_{min}$(e $Å^{-3}$)	1.121/-0.991	0.946/-0.748	0.663/-0.625	0.985/-0.725	0.898/-1.151	1.198/-1.735	0.999/-1.169	1.690/-3.209
